# Drugging the Undruggable: Advances on RAS Targeting in Cancer

**DOI:** 10.3390/genes12060899

**Published:** 2021-06-10

**Authors:** Miriam Molina-Arcas, Amit Samani, Julian Downward

**Affiliations:** 1 Oncogene Biology Laboratory, Francis Crick Institute, London NW1 1AT, UK; julian.downward@crick.ac.uk; 2 Department of Medical Oncology, Imperial College Healthcare NHS Trust, London W2 1NY, UK; 3 Lung Cancer Group, Institute of Cancer Research, London SW3 6JB, UK

**Keywords:** RAS, KRAS-G12C, oncogene, cancer, targeted therapy

## Abstract

Around 20% of all malignancies harbour activating mutations in RAS isoforms. Despite this, there is a deficiency of RAS-targeting agents licensed for therapeutic use. The picomolar affinity of RAS for GTP, and the lack of suitable pockets for high-affinity small-molecule binding, precluded effective therapies despite decades of research. Recently, characterisation of the biochemical properties of KRAS-G12C along with discovery of its ‘switch-II pocket’ have allowed development of effective mutant-specific inhibitors. Currently seven KRAS-G12C inhibitors are in clinical trials and sotorasib has become the first one to be granted FDA approval. Here, we discuss historical efforts to target RAS directly and approaches to target RAS effector signalling, including combinations that overcome limitations of single-agent targeting. We also review pre-clinical and clinical evidence for the efficacy of KRAS-G12C inhibitor monotherapy followed by an illustration of combination therapies designed to overcome primary resistance and extend durability of response. Finally, we briefly discuss novel approaches to targeting non-G12C mutant isoforms.

## 1. Introduction

Mutations in RAS genes occur frequently in solid and haematological malignancies with around 20% of all tumours harbouring a mutation in at least one isoform [1,2]. Lung cancer, which accounts for the most cancer deaths worldwide [3], harbours KRAS mutations in approximately 25% of cases with variation by histology and patient demographic [4,5]. Pancreatic cancer, which has the highest mortality rate of any solid tumour, has an approximately 90% KRAS mutation rate, while colorectal cancer has a rate close to 50% [1]. Across all tumour types, in a large PanCancer analysis, KRAS was the most diverse oncogene, being labelled as a driver mutation in 16 different primary sites [6]. Although mutations in KRAS are the most common pan-cancer, certain tumour types show other isoform predilection. For example, melanoma is enriched for NRAS mutations while HRAS is the most commonly mutated isoform in bladder and head and neck cancers [1].

Most oncogenic mutations in RAS isoforms are missense gain-of-function mutations in codons G12, G13 or Q61 [7]. Different RAS isoforms differ in their frequency of such alterations. In KRAS, G12 and G13 mutations predominate. In contrast, Q61 mutations are rare in KRAS but common in NRAS, while the frequencies in HRAS lie in between the other two isoforms. Furthermore, within a given isoform and codon, the frequency of amino acid substitutions varies by cancer type. For example, in lung cancer, the commonest KRAS G12 mutation is G12C, possibly associated with tobacco smoke exposure [8], while in colon and pancreas cancers, G12D is the commonest. The spectrum of mutations in isoforms and tumour types has been reviewed well, elsewhere [9,10].

RAS proteins are small GTPases that cycle between GDP-bound (inactive) and GTP-bound (active) conformations. GTP-RAS activates several downstream signalling cascades, the most well studied of which are the MAPK (RAF/MEK/ERK) and PI3K (PI3K/AKT/mTOR) pathways. Impaired GTP hydrolysis, resulting in increased flux through downstream pathways, is a key property of oncogenic RAS [11,12]. However, different mutations result in distinct biochemical and structural properties. Differences occur in (a) GTP binding affinity, (b) intrinsic and GTPase-activating protein (GAP)-mediated GTP hydrolysis and (c) effector binding affinity [13]. In fact, analysis of intrinsic GTP hydrolysis rates of various KRAS mutations showed that while G12A, G12R, Q61H and Q61L mutations resulted in 40–80× reduction compared to wild type, G12C was an outlier, with a rate almost comparable to wild type. This is consistent with observations that KRAS G12C isoforms demonstrate rapid GTP–GDP cycling, a phenomenon exploited by recently developed inhibitors [14].

In this review, we discuss initial approaches to target oncogenic RAS, including farnesyl transferase inhibitors and small molecule inhibitors of RAS–effector pathways. We next look at the development of KRAS-G12C inhibitors, a class of molecule in advanced-phase clinical trials. Finally, we briefly discuss emerging approaches in the field.

## 2. Initial Approaches to Target RAS

Early attempts to develop direct RAS inhibitors failed due to the picomolar affinity of RAS for GTP and the lack of suitable hydrophobic pockets which could allow high-affinity binding of small molecules [15]. In the 1990s, a more indirect approach was taken, and efforts focused on the development of inhibitors that targeted RAS’s post-translational processing. RAS proteins are modified post-translationally in order to promote association with the inner face of the plasma membrane where they become active. The first step is prenylation of the C-terminal CAAX motif by a farnesyltransferase (FTase); next, the last three amino acids (AAX) are removed by a RAS-converting enzyme (RCE1); and finally, the carboxyl group of the cysteine is methylated by isoprenylcysteine carboxyl methyltransferase (ICMT) [16]. Several farnesyl transferase inhibitors (FTI) were developed and showed promising pre-clinical results against HRAS-driven cancer models [17]. However, lonafarnib and tipifarnib disappointingly failed in clinical trials against KRAS-mutant-driven cancers. The reason for failure was discovered when isoform-specific differences were studied. KRAS and NRAS can bypass prenylation of the cysteine at the CAAX motif by using a geranylgeranyl isoprenoid [18]. Unlike KRAS and NRAS, HRAS can only be prenylated by FTase; therefore, FTIs can still be useful for the treatment of HRAS-mutant cancers. In fact, the positive results of tipifarnib in a phase II clinical trial for the treatment of HRAS-mutant HNSCC and thyroid cancer (NCT02383927) has recently resulted in a Breakthrough Therapy Designation (BTD) by the FDA for the treatment of patients with recurrent or metastatic HRAS-mutant HNSCC. Dual farnesyltransferase and geranylgeranyltransferase I inhibitors have also been developed; however, these inhibitors were either toxic or failed to achieve a good inhibition of KRAS prenylation [19,20]. In parallel, efforts to inhibit KRAS post-translational modifications have moved to target the downstream processing enzymes, RCE1 and ICMT [21,22]. Recent data show that NRAS is strongly dependent on ICMT activity for efficient trafficking, which would suggest that ICMT inhibitors could be a good therapeutic strategy for NRAS-driven tumours [23]. Another potential target that has been recently identified is the prenyl-binding protein phosphodiesterase δ (PDEδ), which regulates RAS localisation and trafficking [24]. However, these enzymes will likely affect the function of other farnesylated proteins and could result in off-target effects.

## 3. Targeting RAS Pathways

Due to the inherent difficulties in directly targeting RAS or its post-translational modifications, in recent years the most commonly pursued strategy has been to target RAS effector signalling. This has resulted in the development and clinical evaluation of a battery of highly selective inhibitors against downstream effectors of RAS, predominantly the RAF/MEK/ERK and PI3K/AKT/mTOR pathways. The importance of the RAF/MEK/ERK pathway for the survival and proliferation of tumour cells harbouring RAS mutations has been demonstrated in several studies [25,26,27]. Large-scale drug screening approaches using extensive panels of cell lines have shown that RAS mutations are predictors of sensitivity to MEK inhibition [28], which explains why MEK inhibitors have been the most heavily investigated. An analysis of gene expression signatures reflecting RAS pathway activation in lung cancer shows that MEK inhibitory drugs have the strongest selective effectiveness towards RAS pathway active lung cancer cell lines out of a panel of some 500 oncology drugs [29]. Several MEK inhibitors are used clinically, with trametinib, cobimetinib and binimetinib being approved for BRAF-mutant melanoma [30], while selumetinib is also approved for use in children with neurofibromatosis type 1, an example of MEK inhibitor efficacy in tumours with RAS pathway perturbation that is not in RAS itself [31]. However, although MEK inhibitors are effective against BRAF-mutant melanoma and in neurofibromatosis, they have shown only modest or no response in clinical trials in RAS-mutant tumours [32]. An example of the lack of clinical efficacy of MEK inhibitors is the combination of the MEK inhibitor selumetinib plus docetaxel. Although the phase II clinical trial in a KRAS-mutant non-small cell lung cancer (NSCLC) cohort showed promising efficacy, this activity was not confirmed in a phase III randomised study [33,34]. Interestingly, the mechanism of action of the MEK inhibitor can determine the different efficacy between KRAS- and BRAF-mutant tumours; specifically, MEK inhibition that also blocks feedback reactivation of MEK by wild-type RAS enhanced efficacy in RAS-mutant tumours relative to BRAF-mutant tumours [35]. This suggests that a better molecular understanding of the mechanism of inhibition can provide essential information for the design of the next generation of MEK inhibitors [36].

Even more complex has been the study of RAF inhibitors. Surprisingly, in contrast to the efficacy observed in BRAF-mutant melanoma, BRAF inhibitors were found to activate ERK and stimulate growth of RAS-mutant cancers by an effect known as the RAF inhibitor paradox [37,38]. This led to the development of pan-RAF inhibitors and inhibitors that block RAF dimerization, which do not activate the MAPK pathway [39]. However, these inhibitors also showed limited clinical efficacy as a single therapy. Recent data from genetic screens and mouse models have opened up the new possibility of targeting specific RAF isoforms. Using whole-genome CRISPR screens to identify genetic dependencies, CRAF scored as the top essential gene in KRAS-mutant cell lines [40]. These data are supported by the fact that ablation of CRAF, but not BRAF, results in tumour regression of KRAS-mutant lung tumours, whereas total body ablation in adult mice is well tolerated [41]. However, loss of CRAF does not alter MAPK activity and kinase inactive CRAF does not mimic the effects of CRAF ablation in tumours, suggesting a kinase-independent CRAF function [42]. Therefore, although these studies suggest the therapeutic benefit of targeting CRAF, novel therapeutic strategies will be needed.

More recent has been the development of ERK inhibitors [43,44]. However, similarly to MEK and RAF inhibitors, targeting ERK as a monotherapy failed to achieve clinical responses in patients with RAS mutations, although some responses were observed in patients with BRAF-mutant melanoma [45].

The lack of clinical benefit of the existing RAF, MEK and ERK inhibitors as monotherapies could be explained by two main reasons. RAS has multiple downstream effector pathways. Therefore, targeting only MAPK signalling may not be enough for clinical efficacy. On the other hand, inhibition of ERK results in the loss of the negative feedback loops that regulate the activity of the pathway. This causes the reactivation of the MAPK pathway, and in some cases of the PI3K/AKT pathway, mainly due to upregulation of receptor tyrosine kinase (RTK) activity. This suggests that combination therapies with RAF, MEK or ERK inhibitors are needed to achieve an effective response. However, identification of optimal combinations depends not only on the biology of RAS-mutant tumours, but also on the selection of combinations that achieve a strong pathway inhibition with an adequate therapeutic index.

One strategy to achieve potent MAPK inhibition and overcome resistance mechanisms is by vertical inhibition of the RAF/MEK/ERK pathway. This is now extensively used in BRAF-mutant melanoma [46]. For example, the addition of the MEK inhibitor trametinib to the BRAF inhibitor dabrafenib in the metastatic setting improves objective response rate (ORR) from 51 to 64% and three-year overall survival from 32 to 44% [47,48]. Nonetheless, five-year progression-free survival with the combination was 19%, suggesting that the response is not durable in the majority [49]. In RAS-mutant cell lines, combinations of MEK/ERK, MEK/RAF and RAF/ERK inhibitors have shown enhanced antitumour activity [50,51,52] and some clinical trials combining RAF and MEK inhibitors in RAS-mutant cancers are ongoing. However, as indicated above, some tumours may need inhibition of more than one downstream effector pathway. The PI3K/AKT pathway is the second most-studied RAS–effector pathway and its critical role in RAS-driven tumorigenesis has been demonstrated in mouse models [53,54]. Combinations of MEK and PI3K inhibitors have shown promising results in a preclinical model [55]. However, clinical trials observed either a high degree of toxicity or low target inhibition at the maximum tolerated doses [56,57]. Because PI3K activity may be influenced by several upstream pathways, one way to overcome high levels of toxicity could be to suppress PI3K indirectly. Interestingly, PI3K activation in KRAS-mutant lung and colon cells is dependent on insulin-like growth factor 1 receptor (IGF1R) activity and a combination of MEK and IGF1R inhibitors showed synergistic effects both in NSCLC and colorectal cancer (CRC) [58,59]. Combinations with other RTKs have also been proposed in order to block the feedback reactivation of the MAPK pathway [60,61]. However, the RTK activated may depend not only on the tissue of origin but also on other specific cellular attributes. For example, combination of MEK inhibitors with ErbB inhibitors is effective in epithelial NSCLC cells, whereas in mesenchymal cells pathway reactivation is blocked with FGFR inhibitors [62].

Although targeting RTK-driven pathway reactivation is an attractive option, the fact that different, or even multiple, RTKs can be activated after MEK inhibition precludes the selection of a universal combination strategy. An alternative that has been recently proposed is the targeting of the RTK-associated phosphatase SHP2 (encoded by PTPN11). Although the exact biological function of SHP2 is not clear, it is known that SHP2 acts as key node linking RTK–RAS signalling, probably acting as a scaffold protein which binds GRB2 and SOS1, therefore promoting the activation of RAS proteins by stimulating GDP for GTP exchange [63,64]. Ablation of SHP2 inhibits tumour progression in NSCLC and PDAC mouse models and similar results have been obtained using allosteric SHP2 inhibitors [65]. Interestingly, only those cells bearing KRAS mutations that maintain intrinsic GTPase activity are sensitive to SHP2 inhibition. Cells harbouring KRAS-G12C mutations, which have high intrinsic GTPase activity, are generally the most sensitive to SHP2 inhibition, whereas cells bearing mutations in G13 or Q61 are insensitive [66]. SHP2 inhibitors also prevent adaptative resistance to MEK inhibitors and combinations of MEK plus SHP2 inhibitors produce tumour regression in several RAS-driven tumours [67]. Currently there are three SHP2 inhibitors in phase I/II clinical trials, RMC-4630, JAB-3068 and TNO155. RMC-4630 is also being tested in combination with the MEK inhibitor cobimetinib (NCT03989115). Another mechanism to inhibit RTK-mediated activation is by blocking the activity of the RasGEF SOS1. SOS1 inhibitors have also been developed recently [68,69]. Although they are not as potent as a monotherapy compared to SHP2 inhibitors, they also reduce the feedback reactivation induced by MEK inhibitors and increase the sensitivity to MEK inhibition [69]. Currently, the SOS1 inhibitor BI-1701963 is in a phase I clinical trial as a monotherapy or in combination with the MEK inhibitor trametinib (NCT04111458).

Finally, other strategies designed to improve the efficacy of MEK inhibitors involve targeting proteins that are outside the RAS pathway. Targeting the cyclin-dependent kinase 4 (CDK4) was shown to be synthetically lethal in KRAS-mutant NSCLC [70]. These data prompted analysis of the combination of MEK inhibitors with CDK4/6 inhibitors, showing preclinical efficacy in several models of KRAS-mutant cancer [71,72]. Several CDK4/6 inhibitors have been approved for metastatic breast cancer [73] and phase I/II trials combining MEK or ERK inhibitors with the CDK4/6 inhibitor palbociclib in KRAS-mutant tumours are ongoing. Another approach has been to increase the apoptotic response to MEK inhibition. MEK inhibitors usually result in cytostatic effects; however, apoptosis can be increased when combined with inhibition of the anti-apoptotic BH3 family protein BCL-X_L_ or genetic ablation of BCL-2 [74]. Based on these pre-clinical findings, a clinical trial combining the MEK inhibitor trametinib and the BCL-2/BCL-X_L_ inhibitor navitoclax is ongoing (NCT02079740). Several studies have also indicated that after MEK inhibition, KRAS-mutant tumours are more dependent on autophagy and that co-targeting MEK and autophagy results in a synergistic response [75,76]. One study tested this hypothesis in a patient with metastatic PDAC by combining trametinib with hydroxychloroquine. The treatment resulted in a clear reduction in tumour burden [76]. Based on these preliminary data, several trials combining MEK inhibitors and hydroxychloroquine in KRAS and NRAS mutant tumours have started.

## 4. Direct Targeting of KRAS: G12C Inhibitors

As discussed above, G12C-mutant KRAS can be distinguished by a unique biochemical property—its relatively preserved intrinsic GTP-hydrolysis rate. In addition, the mutant cysteine renders the molecule susceptible to specific, and irreversible, binding with cysteine-reactive small molecules. These properties were exploited by Shokat and colleagues who screened 480 tethering compounds against KRAS-G12C [77]. They discovered two in particular which reacted in a manner independent of ambient GDP concentration, indicating an allosteric effect, identified as binding to the switch-II pocket (P2), which lies close to the effector-binding switch-II region. Binding occurred in the inactive, GDP-bound, state. In the GTP-bound state, residues from P2 obscure the pocket, precluding binding. Once drug bound, RAS function is impaired; nucleotide affinities are altered (inducing a preference for GDP over GTP) and SOS-catalysed nucleotide exchange is blocked, effectively ‘trapping’ the protein in the inactive state, impairing effector interaction [14,78].

Continuing to exploit P2 for drug binding, ARS-853, a compound with robust cellular activity, was developed [14]. Modelling the dose/time dependence of KRAS-engagement by drug suggested that KRAS-G12C cycles rapidly between the GTP- and GDP-bound state, consistent with previous work showing its much faster intrinsic hydrolysis rate compared to other mutants [13]. This explains the paradox that the ARS-853 robustly inhibits KRAS signalling despite specificity for the GDP-bound state, given the abiding assumption that oncogenic RAS is predominantly GTP bound.

Treatment of KRAS-G12C-mutant cell lines with ARS-853 resulted in pathway inhibition downstream of KRAS. However, there was heterogeneity in the depth and durability of effect across different cell lines, suggesting that factors beyond the G12C mutation may modify sensitivity. In terms of viability, some lines were insensitive to the drug, and the same cells showed lack of response to genetic ablation of KRAS, suggesting KRAS independence in spite of the G12C mutation. In 3D culture, however, all lines showed robust inhibition of proliferation following treatment [14], demonstrating heterogenous KRAS dependency with increased sensitivity to KRAS inhibition in 3D vs. 2D culture. ARS-1620 followed on from ARS-853, being modified for improved in vivo efficacy. Sensitivity of lines again varied between 2D and 3D culture; however, the latter predicted in vivo efficacy, correlating well with drug response in a panel of xenograft models [79]. Following this, further compounds were developed with in vivo activity against KRAS-G12C [80,81]. These showed robust activity in pre-clinical models, paving the way for clinical studies.

### 4.1. Clinical Data with KRAS-G12C Inhibitors

There are several active and recently completed clinical trials involving KRAS-G12C inhibitors (Table 1). Of these, three are active phase III trials involving two compounds, AMG 510 (sotorasib) and MRTX849 (adagrasib) [80,81]. Both compounds have produced robust pre-clinical evidence.

In vitro treatment with AMG 510 resulted in inhibition of phospho-ERK (p-ERK) and phospho-S6 (p-S6) and increased cleaved caspase-3 specifically in KRAS-G12C-mutant cells [80]. Again, there was heterogeneity in the concentration and time-dependent effects across the G12C-mutant repertoire with around a 10-fold difference in both p-ERK and viability IC_50_ values between cell lines. MRTX849 too was able to inhibit in vitro RAS function and G12C-mutant cell viability with IC_50_s in the nanomolar range [81]. Pre-clinical in vivo models for AMG 510 showed maximal p-ERK inhibition 2–4 h after treatment, an effect that was sustained for 48 h consistent with covalent inhibition of the KRAS-G12C protein (t_1/2_ = 20–24 h). Tumour regression also occurred. This was dose-dependent, with an increased proportion of complete regressions at a higher dose, possibly correlating with the degree of p-ERK inhibition [80]. Similar effects were seen with MRTX849, which exhibited tumour responses in most of the models tested [81].

Following these promising pre-clinical results, early-phase clinical evidence has also been encouraging. The KRYSTAL-1 study (NCT03785249) is a phase I/II study which is recruiting patients with pre-treated NSCLC, CRC and other advanced solid tumours. Arms include MRTX849 monotherapy as well as combinations with pembrolizumab, cetuximab and afatinib [82,83]. In one analysis of 110 patients (79 NSCLC), 30% of patients developed grade III or IV treatment-related adverse events (TRAEs) at the phase II dose of 600 mg [84]. By way of comparison, the grade 3 TRAE rate from a similar study using a MEK inhibitor was 40%, suggesting better tolerability for G12C-targeted therapy [85]. Efficacy data from 65 evaluable NSCLC patients showed an objective response rate (ORR) of 45%. A recent update showed that for the 12 patients treated with 600 mg BD in the phase I portion of the trial, 4/6 who showed a partial response remained on treatment for >11 months with an ongoing response at the time of reporting, suggesting that the response to drug is prolonged, at least in a subset [86]. In those with CRC, confirmed ORR was only 17 (3/18 patients) and 67% (4/6, one unconfirmed) in those with other solid tumours including pancreatic, ovarian (unconfirmed), endometrial and cholangiocarcinoma [87]. The reasons for the disparity between NSCLC and CRC efficacy parameters are unknown but may be due to enhanced EGFR wild-type signalling in the latter, as discussed below [88].

KRAS mutations in NSCLC often co-occur with mutations in tumour-suppressor genes including TP53, STK11 and KEAP1 [89]. An exploratory analysis of co-mutations in a clinical trial cohort showed a trend toward a higher response rate in STK11-co-mutated tumours (64 vs. 33% in WT), while, conversely, KEAP1 and TP53 co-mutations were not predictive of response [84]. While these data are exploratory, it is known that STK11-mutated tumours have a poor response to immunotherapy [90] and KRYSTAL-1 data open the possibility of a biomarker-driven effect in this difficult-to-treat subgroup. Following the success of KRYSTAL-1, the phase III KRYSTAL-12 study randomising pre-treated patients with NSCLC to MRTX849 600 mg vs. docetaxel has begun recruiting.

Separately, the CodeBreak 100 trial is a large study with several phase I/II arms investigating AMG 510 as monotherapy or in combination with anti PD-1/PD-L1 therapy (NCT03600883). The phase I monotherapy portion of CodeBreak 100 treated 129 patients of whom 59 had NSCLC, 42 CRC and 28 had other tumour types [91,92,93]. Patients were generally heavily pre-treated. The drug was well tolerated with a grade 3/4 TRAE rate of 11.6%, reflective of the specificity seen in the pre-clinical data. The ORR in NSCLC was 32.2% but only 7.1% in CRC (3/42 patients). Therefore, similar to MRTX849, response rates are higher in NSCLC compared to CRC. Partial responses (PRs) were also seen in patients with pancreatic, endometrial, appendiceal and melanomatous skin cancer, indicating the potential for therapeutic responses in selected patients across other tumour types [93].

Early results from the phase II portion of the AMG 510 monotherapy arm of CodeBreak 100, treating only patients with advanced NSCLC, were recently reported [94]. Of 123 patients with evaluable disease, the ORR was 37.4% and there was a disease control rate (the sum of ORR and stable disease) of 81%. The median duration of response was 10 months. TRAEs at this dose occurred in 20.6% and were similar to phase I results. CodeBreak 200, a phase III study investigating AMG 510 versus docetaxel in previously-treated NSCLC has also begun recruiting with results awaited [95]. However, the success of the phase II portion of CodeBreak 100 led to FDA fast-track designation, a programme designed to expedite the development and regulatory review of potentially significant medicines [96]. This has resulted in formal FDA approval of sotorasib in May 2021 for the treatment of advanced or metastatic KRAS-G12C-mutated NSCLC, becoming the first drug targeting KRAS to be approved [97].

Two other G12C inhibitor trials opened for recruitment pre-2020: JNJ-74699157 and LY349934. The latter was terminated due to unexpected toxicity. This highlights the fact that despite the mutant-specific nature of inhibitors, toxicity can still occur, perhaps due to off-target effects on other cysteine-containing proteins despite reassuring in vitro specificity. Newer inhibitors are also in early-phase testing. D-1533, GDC-6036 and JDQ443 trials are all recruiting. Yet, other inhibitors are being explored pre-clinically, including inhibitors from Boehringer Ingelheim and Revolution Medicines. In contrast with the similar approach used by most of the companies, Revolution Medicines has used a completely different strategy to covalently inhibit KRAS-G12C in the active state (GTP bound). These molecules are covalent tri-complex inhibitors where the compound mediates the binding of KRAS-G12C with the chaperone cyclophilin A, preventing the binding of RAS effector proteins [98,99].

### 4.2. Combination of KRAS Inhibition with Targeted Therapy

As with other targeted therapies, resistance to KRAS-G12C inhibitor monotherapy will limit clinical efficacy and efforts are underway to understand resistance mechanisms. The development of G12C inhibitor combinations has benefited from prior knowledge of MEK inhibitor combinations. However, with a broader therapeutic window, it is likely that the mutant-specificity of G12C inhibition will enhance synergy and mitigate toxicity compared to MEK inhibitor combinations.

**Figure 1:**
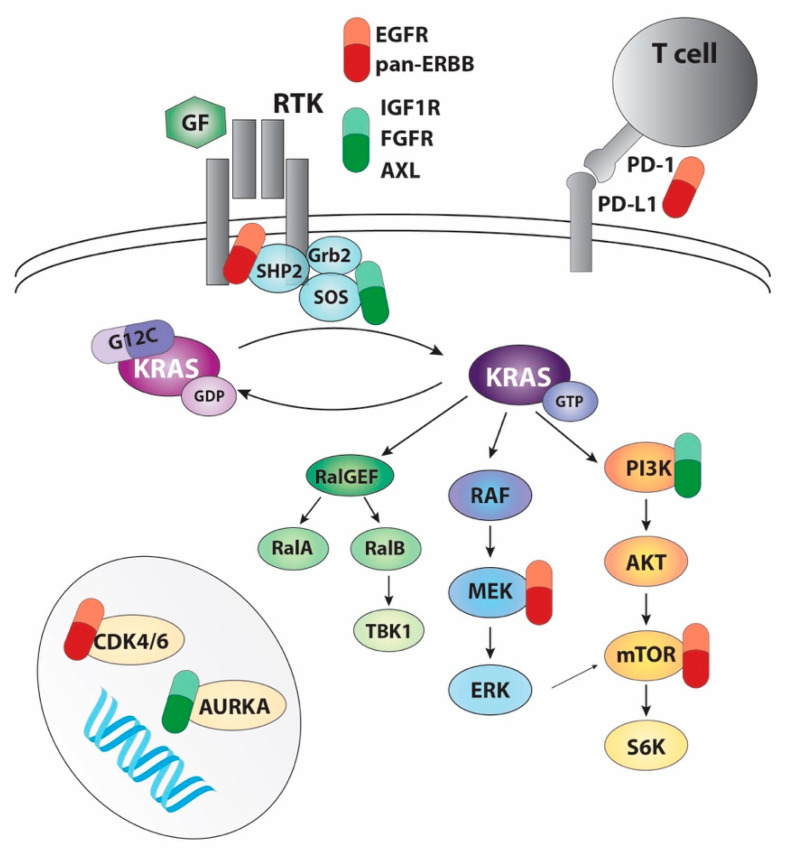
General overview of the combinations that have been proposed to increase the efficacy of KRAS-G12C inhibitors. Targets in red are being tested in clinical trials (see Table 2). Targets in green have been validated in pre-clinical studies.

As alluded to previously, sensitivities of cell lines to KRAS-G12C inhibition vary up to 100-fold in vitro [81]. These sensitivities are not correlated with binding to target; following drug binding, electrophoretic mobility shift IC_50_ values were similar across lines, suggesting that even resistant lines undergo robust target engagement [81]. Inhibition of ERK and S6 phosphorylation, however, did vary, and correlated with sub-maximal viability response in culture [81]. This suggests that maximising inhibition of these targets could augment response. Furthermore, evidence that these drugs bind mostly to GDP-bound KRAS and that KRAS-G12C cycles rapidly between active and inactive conformations suggests that modification of cycling kinetics through targeting upstream components of the RAS cascade could yield benefit. Additionally, selective and unbiased CRISPR screens can help identify synthetic-lethal partners to RAS. We briefly discuss some of these approaches below (Figure 1).

In order to understand KRAS dependency, one study looked at signalling in pancreatic cell lines following genetic ablation of KRAS. This was tolerated in a subset and while these did not display increased YAP1 activity or sensitivity, as had previously been shown in KRAS-deficient models [100], they did have high basal PI3K/AKT pathway activity and showed increased sensitivity to pan-PI3K and mTOR inhibition [101]. These results suggest that hyperactivation of the PI3K/AKT pathway, possibly driven by upstream RTK signalling, can facilitate oncogenic KRAS independence. Further evidence for synergy between RAS and PI3K blockade comes from a study using G12C-mutant NSCLC lines [102]. Four of the twelve lines tested showed little response to ARS-1620 on downstream signalling at 48 h despite sustained reduction in KRAS-GTP. Across the lines, inhibition of p-AKT and p-S6 levels at shorter time points was also variable with only the most sensitive lines showing suppression. To identify synergies, the authors performed a drug screen. While RTK inhibitor combinations produced isolated strong synergies, these were not homogenous across lines, unlike PI3K/AKT-axis targeting, which was more consistent in their effect. In line with this, suppression of viability and tumour growth was more consistent when ARS-1620 was combined with GDC0941 (pan-PI3K inhibitor), compared to afatanib (EGFR/ErbB inhibitor), in different ARS-1620 monotherapy-resistant models. A recent study in support of similar synergy proposed the combination of KRAS-G12C plus mTORC1/2 inhibitors which reliably suppressed pERK and pAKT and induced cell death in pancreatic cancer models [103].

While combined PI3K and KRAS-G12C inhibition appears broadly effective, the studies above also suggest RTK co-inhibition could work, although they highlight the heterogeneity of optimal synergy between different cell lines. To overcome this, SHP2 inhibitors have been proposed as a way to delay adaptive resistance to G12C inhibitors [104]. In one study, ARS-1620 treatment of G12C-mutant lines resulted in a rapid rebound of ERK phosphorylation due to an increase in GTP-bound wild-type RAS. The authors showed that this was partly due to a heterogenous adaptive increase in phosphorylation of several RTKs (EGFR, HER2, FGFR and c-MET) across the cell lines, resulting in activation of wild-type RAS. In each line, inhibition of a specific RTK was able to markedly decrease viability but no inhibitor was effective across the board. SHP2 inhibition, however, suppressed adaptive resistance across all lines and enhanced suppression of active RAS [104]. Combinations of SHP2 and KRAS-G12C inhibitors led to deeper and more sustained inhibition of both KRAS and total RAS-GTP than either agent alone. A more recent comprehensive study using SHP2 and KRAS-G12C inhibitor combinations in vitro and in vivo in both xenograft and syngeneic PDAC and NSCLC models showed a benefit of the combination. This encompassed both tumour regression and immune tumour microenvironment remodelling, as discussed later [105]. A separate study also supported the utility of SHP2 inhibitors in combination with G12C blockade, albeit via a different mechanism [106]. This team used single-cell sequencing of three KRAS-G12C-mutant lung cancer lines treated with ARS1620 for variable lengths of time. They found that some cells were able to circumvent the effects of treatment through synthesis of new KRAS-G12C protein in conjunction with enhanced upstream signalling. Although this mechanism of adaptive resistance differed compared to other studies (synthesis of new KRAS-G12C vs. upregulation of wild-type RAS-GTP), upstream signalling to maintain flux through the MAPK pathway remained a common theme. Overall, these studies suggest that KRAS inhibition, similar to MEK blockade, can lead to feedback activation of RTKs and drive reactivation of RAS downstream pathways. The combination with SHP2 inhibitors offers a potential opportunity to block multiple RTKs and therefore maximize the benefit of the combination. Moreover, SHP2 inhibitors can increase KRAS-GDP occupancy, which would enhance the efficacy of KRAS-G12C inhibitors [105]. Similar potentiation of efficacy of KRAS-G12C inhibitors can be achieved using SOS1 inhibitors, although this combination strategy has not been extensively studied [69].

Although heterogeneity of RTK activation may preclude the use of specific RTK inhibitors as a general synergistic mechanism, some tumours may display characteristics suggestive of the most likely kinase to mediate resistance. For example, EGFR signalling was found to be the dominant mechanism of resistance in one study on colorectal cancer [88]. Indeed, the combination of the KRAS-G12C inhibitor MRTX1257 and the EGFR antibody cetuximab is being explored in a phase III trial in patients with advanced colorectal cancer (NCT04793958). Furthermore, gene expression signatures may help stratify patients for optimal RTK inhibitors [107]. In both cell lines and human G12C-mutant tumours, those with an epithelial signature had high basal ErbB2/3, while those with a mesenchymal signature had high basal FGFR2 and AXL. Thus, combination RTK/G12C inhibition has potential to be a powerful therapy in biomarker-selected patients.

The studies above focussed on targeting either upstream or downstream of RAS. A third approach entails simultaneous targeting of both [108]. Utilising a comprehensive drug screen, inhibitors of IGF1R showed selectivity for the RAS-mutant genotype [58]. Combining these, MEK and mTOR inhibitors showed profound inhibition of RAS downstream pathways and suppression of viability across cell lines. In patients, the combination of MEK and mTOR inhibition is toxic [109], but the advent of G12C-specific inhibitors could circumvent this. In a recent study [108], the combination of ARS-1620 with mTOR and IGF1R inhibitors enhanced inhibition of proliferation compared to dual targeting strategies using both xenografts and an immunocompetent KRAS-G12C mouse model. A similar strategy was employed in a separate study, which discovered induction of epithelial-to-mesenchymal transition as a resistance mechanism to KRAS inhibition and highlighted IGFR-IRS1 regulation of PI3K as key to this resistance [110]. The authors were able to achieve synergy by combining SHP2 and PI3K inhibition with KRAS-G12C blockade.

Beyond the RTK–RAS–MAPK/PI3K pathways, CRISPR and similar screens have the potential to identify novel synergistic partners. In a focussed CRISPR screen of 400 genes in cells treated with MRTX849 treatment [81], classic RAS-related genes such as MYC and mTOR pathway genes resulted in loss of fitness, as did cell cycle genes. In the same study, a focussed library of 70 small-molecule inhibitors found synergy between RAS-pathway inhibition and CDK4/6 inhibition. Combining MRTX849 with palbociclib caused near-complete inhibition of Rb phosphorylation in contrast to the partial inhibition caused by MRTX849 monotherapy and resulted in tumour regression in five different tumour xenograft models [81]. Combinations of KRAS-G12C and CDK4/6 have also been proposed in two other studies [111,112]. Interestingly, Hallin and co-authors propose that this combination could be more efficient in models with CDKN2A homozygous deletion, which suggests that some combinations would be more beneficial in a subset of patients.

Combination strategies to prolong sensitivity to KRAS-G12C inhibitors are already in early-phase clinical trials (Table 2). Several trials in combination with SHP2 inhibitors including CodeBreak 101, KRYSTAL-2 and JDQ443 are recruiting. A combination trial with a SOS1 inhibitor and MRTX849 is due to start soon. Although several combinations with RTK inhibitors have been proposed, only targeting of ErbB family members is being tested in clinical trials, including a pan-ErbB or EGFR inhibitor (CodeBreak 101), cetuximab or afatinib (KRYSTAL-1) and cetuximab versus chemotherapy in the phase III KRYSTAL-10 trial in pre-treated colorectal cancers. Downstream targeting with a MEK inhibitor is being investigated in CodeBreak 101 (with or without combination EGFR inhibitor), while targeting of the PI3K/mTOR pathway with an mTOR inhibitor is planned in the same trial. Finally, combinations targeting cell cycle with CDK4/6 inhibitors are also being planned in different clinical trials.

### 4.3. Combination of KRAS Inhibition with Immunotherapy

Although clinical results with KRAS-G12C inhibitors are promising, drugs targeting oncogenic drivers usually result in a rapid development of resistance after promising initial responses. On the other hand, checkpoint inhibitor therapy, which is the standard-of-care treatment for patients with advanced NSCLC who lack a driver mutation [113], results in potential durable responses in a subset of patients [114]. Despite this, many patients do not respond, or relapse after a period of response. KRAS-mutant lung cancers are often smoking-associated and have high tumour mutational burden and PD-L1 expression. Although they possess this immunogenic substrate which could sensitise to immunotherapy, oncogenic KRAS is capable of inducing an immunosuppressive tumour microenvironment, thus preventing optimal response to checkpoint blockade [115,116]. If inhibition of oncogenic KRAS can reduce these tumour evasion mechanisms, combination KRAS-G12C inhibition with checkpoint blockade could circumvent some limitations of either strategy alone.

KRAS-G12C inhibition has been shown to have effects on tumour-intrinsic mechanisms of immune subversion [117]. In five human xenograft models of NSCLC, inhibition of oncogenic KRAS induced downregulation of CXCL1, CXCL8, NT5E and VEGFA mRNA, downregulation of CD274 (PD-L1) mRNA and upregulation of class I MHC. In a syngeneic model (CT26, colon carcinoma), treatment for 4 or 8 days induced a reduction in myeloid-derived suppressor cells, an increase in macrophage ‘M1′ polarisation, recruitment of dendritic cells and increased T-cell number (including T-regulatory cells). Although MRTX849 treatment induced rapid tumour regression, this was transient in 9/10 mice. Conversely, when combined with an anti-PD-1 antibody, regression was durable in 6/10 mice who were also immune to subsequent tumour rechallenge. Furthermore, the magnitude and duration of response to MRTX849 treatment of CT26 tumours in immune-compromised mice was diminished relative to wild-type mice. These data suggest that even as monotherapy, therapeutic MRTX849 efficacy was partially due to the generation of an adaptive anti-tumour immune response.

In a separate study, long-term cures induced by AMG 510 in a subcutaneous CT26 model were also suggested to rely on anti-tumour immunity [80]. Similar to the experiments with MRTX849, only 1/10 mice had durable regression with AMG 510 alone; however, combination with anti-PD-1 therapy resulted in complete responses in 9/10 mice. Again, KRAS-G12C inhibitor treatment resulted in an increase in T cells, CD103^+^ cross-presenting dendritic cells and macrophage infiltration after just 4 days of treatment. MEK inhibitor treatment in the same model did not have this effect and furthermore, MEK inhibition but not AMG 510 was shown to impair T-cell proliferation in a co-culture system in vitro. This may partly explain the lack of clinical success when combining MEK or BRAF inhibition with immunotherapy, while also suggesting that use of mutant-specific inhibitors such as AMG 510 could overcome this.

Further evidence for the potential of KRAS-G12C inhibition to reduce the immunosuppressive tumour microenvironment produced by oncogenic KRAS and potentially sensitise tumours to immune checkpoint blockade came from another study using the 3LL ΔNRAS murine lung carcinoma line [118]. Using imaging mass cytometry, significant changes in tumour immune contexture induced by KRAS inhibition were clearly evident. These included infiltration of CD8^+^ cells which displayed PD-1 upregulation, especially when in close proximity to tumour cells, suggesting tumour-driven activation. While these studies were performed in lung cancer, similar effects were seen in a mouse model of Kras^G12C^/Tp53^R172H^ pancreatic cancer. Treatment with ARS-1620 increased the percentage of T cells while decreasing granulocytic MDSCs [105]. KRAS-G12C inhibition also increased T-cell chemoattractant cytokines, such as CXCL9-11, an effect that was also seen with AMG 510 [80]. These changes in the tumour microenvironment were more pronounced in SHP2/KRAS-G12C dual inhibitor-treated mice than those treated with ARS-1620 monotherapy. Moreover, these effects were enhanced yet further when anti-PD-1 therapy was also included, raising the intriguing possibility of using a triple combination of dual-targeted therapy with checkpoint blockade in the clinic. In a study in colon cancer mouse models, oncogenic KRAS was found to promote MDSC migration into the tumour microenvironment by repressing IRF2, leading to high expression of CXCL3 [119]. In mouse models of pancreatic ductal adenocarcinoma, KRAS-dependent evasion of NK- and B-cell responses was found to be due to MYC-mediated repression of the Type I interferon pathway [120]. Interplay between RAS signalling and suppression of interferon pathways was also implicated in a mouse KRAS-mutant lung cancer model that was engineered to have increased tumour mutational burden; KRAS-G12C inhibition reprogrammed the tumour microenvironment to favour anti-tumour immune responses with increased antigen presentation, cytokine production, interferon signalling, immune cell infiltration and T-cell activation [121].

Based on this pre-clinical evidence, trials combining KRAS-G12C inhibition and immunotherapy are underway (Table 2). The CodeBreak 101 trial, a multi-arm phase I trial, is investigating several combinations involving AMG 510, including a combination arm with checkpoint blockade. The combination of MRTX849 and pembrolizumab cleared the dose-limiting toxicity observation period in the phase I/II KRYSTAL-1 trial and is now formally entering the phase II stage, as the KRYSTAL-7 trial where MRTX849 will be combined with pembrolizumab in two arms, according to PD-L1 tumour proportion score. Finally, GDC-6036 is being combined with atezolizumab (anti-PD-L1) in a phase Ia/Ib trial and JDQ443 with the anti-PD-1 antibody spartalizumab in a phase Ib/II trial that opened in February 2021.

## 5. Novel Strategies to Target RAS

Although the development of KRAS-G12C inhibitors is a great advance for the treatment of patients carrying this mutation, there is still a need to target other KRAS mutations. However, other approaches will be needed, as these proteins will lack the presence of the reactive cysteine that allows the covalent binding of the inhibitor. Mirati Therapeutics has developed a selective G12D inhibitor (MRTX1133) that targets both active and inactive states, although the details of the mechanism have still not been published [122]. A cyclic peptide that selectively blocks KRAS-G12D in the GTP-bound state has also been identified [123]. However, this compound still needs further development to increase cell permeability. Revolution Medicines has used the tri-complex technology platform described above to develop other RAS inhibitors. The most advanced is a RAS^MULTI^(ON) inhibitor, active against tumours harbouring KRAS-G12D and KRAS-G12V mutations, although their portfolio also includes KRAS-G12D- and KRAS-G13C-selective inhibitors [99].

Due to the wide-range of potential targets, small-interfering RNAs have promising therapeutic potential as mutant-specific RAS inhibitors. Although their short serum and intra-cellular half-life can limit efficacy, local administration may help concentrate siRNA in the tumour microenvironment. Such an approach is employed in an ongoing phase II trial (NCT01676259), where patients with pancreatic cancer receive intratumoural injection of a KRAS-G12D-specific siRNA in a biodegradable matrix (allowing prolonged drug release) in combination with systemic chemotherapy [124].

Mutant-specific targeting is also possible through the use of adoptive cellular therapy (ACT). One approach involves extraction of tumour-infiltrating lymphocytes (TILs) followed by selection, expansion and infusion back into the patient. This was used in a patient with metastatic colorectal cancer, where infusion of KRAS-G12D-specific CD8^+^ TILs resulted in regression of all seven lung lesions [125]. A solitary progressing lesion 9 months later was found to have HLA-C^*^08:02 loss, suggesting a specific mechanism of escape to evade ACT-driven immunity. Another approach involves engineering T cells with RAS-specific T-cell receptor (TCR) clonotypes. As a proof-of-concept, serum from patients with NSCLC was screened for neoantigen-reactive T-cell clones. [126]. One patient had CD4^+^ reactivity from blood (but not tumour) to the KRAS-G12V-mutant allele. Donor lymphocytes were transduced with the relevant TCR clones and the engineered cells demonstrated much higher affinity for KRAS-G12V-pulsed target cells relative to those pulsed with wild-type KRAS. At the very least, these studies provide proof-of-concept that mutant RAS alleles can be immunogenic in humans and suggest the potential for subclinical anti-RAS immune responses to be augmented with ACT.

A related approach involves the use of RAS-specific vaccines. One approach involves the intradermal injection of peptides along with an immune adjuvant. In a phase I/II clinical trial of 48 patients with pancreas cancer, intradermal administration of mutant RAS peptides along with a granulocyte-macrophage colony-stimulating factor (GM-CSF) adjuvant, to activate dendritic cells, elicited evidence of RAS-specific cellular immunity associated with a modest improvement in survival [127]. In a separate pre-clinical strategy, to enhance cellular immunity, common oncogenic KRAS variants were conjugated to DNGR-1 antibodies, thus facilitating uptake by cross-presenting dendritic cells. Mice vaccinated either prophylactically or therapeutically with such conjugates demonstrated impaired tumour growth in KRAS-mutant lung cancer models [128]. Other vaccine approaches include the use of mRNA-encoding neoepitopes for common KRAS mutations or administration of dendritic cell vaccines. Several phase I and II clinical trials using different vaccination approaches, in combination with systemic therapies, are underway (NCT03948763, NCT04117087, NCT03592888). A different approach has been the use of oncolytic viruses which specifically target cancer cells with activated RAS signalling. Although currently there are no clinical trials ongoing, novel oncolytic viruses are being developed and may be a viable clinical option in the future [129,130].

For years, scientists have also tried to identify targets that are essential for the survival of cells carrying RAS mutations. Although these synthetic-lethal screens identified promising targets, results have not been translated into clinical advances [131]. A different approach taken more recently is to study how mutant KRAS alters different cancer hallmarks, including evasion of the immune system and dysregulation of cellular energetics [132,133]. Elucidating these mechanisms can result in novel therapeutic strategies to target the vulnerabilities of RAS-driven tumours.

## 6. Future Perspectives and Conclusions

After years considering RAS as undruggable, the development of KRAS-G12C specific inhibitors has resulted in a renewed optimism and scientists have engaged again in the crusade of targeting RAS. The regulatory approval of KRAS-G12C inhibitors, almost forty years after the discovery of RAS, will change the treatment landscape of patients carrying this mutation. However, early clinical data and the previous experience with other targeted therapies, such as BRAF and EGFR inhibitors, indicate that the battle has not been won and that there are still many challenges ahead.

It is expected that these inhibitors will have a limited efficacy as monotherapies; therefore, combination strategies will be needed. As has been discussed above, several combination strategies have been proposed and some of them are being tested in clinical trials. However, the main challenge will be to determine which combination strategies will work best for each type of tumour. Vertical pathway inhibition targeting downstream or upstream nodes of the RAS pathway could be an effective therapeutic strategy for those tumours that show KRAS dependency, whereas tumours with reduced KRAS dependency could require co-targeting of other pathways. Therefore, there is a need to identify biomarkers that can determine which patient can benefit from each strategy. Moreover, this will likely need to be done for each tumour type. Early clinical data with KRAS-G12C inhibitors show that colorectal cancer is more refractory to KRAS inhibition than lung cancer, indicating that a different approach may be needed for this type of tumour.

One of the major challenges ahead is the identification of mechanisms of resistance and how they might be countered. A recent article analysing the cell-free DNA from one patient resistant to MRTX849 identified the presence of several mutations in genes of the RAS pathway, including one mutation in KRAS that affects the binding of the inhibitor [98]. Awad and colleagues extended the analysis to forty patients and identified multiple concurrent alterations in the RAS pathway in six patients [134]. Interestingly, two of the patients had histological transformation from adenocarcinoma to squamous carcinoma, while in 60% of the cases the mechanism of resistance was not identified. These are still early days and analysis of more patients is needed in order to identify the possible mechanisms of resistance. However, the initial data indicate that this will be a complex question to address, as multiple mechanisms of resistance appear possible.

Progress during the development of KRAS-G12C inhibitors and better understanding of the cycling conformations of RAS proteins have aided the identification of inhibitors targeting other RAS alleles. Drugs targeting KRAS-G12D, one of the most prevalent mutations, are the most advanced, including Mirati’s inhibitor MRTX1133, which is progressing towards human studies. Pan-RAS-specific drugs are also being developed in order to target tumours harbouring mutations that do not have a specific inhibitor, with such compounds publicly disclosed from Revolution Medicines and Boehringer Ingelheim. However, this type of inhibitor will face the extra challenge of possible toxicity caused by blocking RAS activity in non-tumoral cells.

These are exciting times for RAS research, as the development of RAS inhibitors is also helping to better understand RAS biology. The integration of knowledge obtained from both basic and clinical research is what has taken us to this point and what will allow us to, finally, remove the ‘undruggable’ label from RAS.

## Figures and Tables

**Table 1:** KRAS-G12C inhibitor trials with monotherapy arms and pre-clinical compounds.

Drug	Company	Trials	Phase	Date Opened	Population
AMG 510	Amgen	CodeBreak 105	I	Dec-19	AST ^1^
		CodeBreak 100	I/II	Aug-18	AST, NSCLC ^2^
		NCT04625647	II	Sep-21	NSCLC
		CodeBreak 200	III	May-20	NSCLC
MRTX849	Mirati	KRYSTAL-1	I/II	Jan-19	AST
		KRYSTAL-12	III	Jan-21	NSCLC
JNJ-74699157 ^3^	Wellspring/Janssen	NCT04006301	I/II	Jul-19	AST
LY3499446 ^4^	Eli Lily	NCT04165031	I/II	Nov-19	AST
GDC-6036	Roche/Genentech	NCT04449874	Ia/Ib	Jul-20	AST
JDQ443	Novartis	NCT04699188	Ib/II	Feb-21	AST
D-1553	InventisBio	NCT04585035	I/II	Feb-21	AST

Ongoing and completed trials with one or more arms of KRAS-G12C inhibitor monotherapy. All trials recruited patients with KRAS-G12C mutations and unresectable locally advanced or metastatic tumours. AST = advanced solid tumours, NSCLC = non-small cell lung cancer. ^1^ Subjects of Chinese ancestry only; ^2^ One arm for untreated patients only; ^3^ Completed July 2020. Only 10 patients recruited; ^4^ Terminated due to an unexpected toxicity finding.

**Table 2:** KRAS-G12C inhibitor trials with combination arms.

Drug	Trials	Phase	Combinations
AMG 510	CodeBreak 100	I/II	Anti-PD-1
	CodeBreak 101	Ib	Anti-PD-1, anti-PD-L1 MEKi +/− EGFRi, EGFRi +/− chemo, CDK4/6i, mTORi, SHP2i, pan-ErbBi
MRTX849	KRYSTAL-1	I/II	Pembrolizumab (anti-PD-1), cetuximab (EGFRi), afatinib (ErbBi)
	KRYSTAL-2	I/II	TNO155 (SHP2i)
	KRYSTAL-7 ^1^	II	Pembrolizumab
	KRYSTAL-10 ^2^	III	Cetuximab
GDC-6036	NCT04449874	Ia/Ib	Atezolizumab (anti-PD-L1), bevacizumab (anti-VEGF), cetuximab (EGFRi), erlotinib (EGFRi)
JDQ443	NCT04699188	Ib/II	TNO155 (SHP2i), spartalizumab (anti-PD-1), TNO155 + spartalizumab
D-1553	NCT04585035	I/II	Several ^3^

^1^ Only patients with NSCLC; ^2^ Only patients with CRC; ^3^ Combination therapies are not detailed in the clinical trial.

## Data Availability

Not applicable.
